# A terahertz graphene-metamaterial sensor for highly sensitive detection of trace pesticide metaldehyde

**DOI:** 10.1371/journal.pone.0338331

**Published:** 2025-12-12

**Authors:** Dazhi Zhang, Xinlei Ruan, Maosheng Yang, Honglai Liu, Xiaobing Li, Peipei Li

**Affiliations:** 1 School of Chemical Engineering & Technology, China University of Mining and Technology, Xuzhou, Jiangsu, P. R. China; 2 Xuzhou College of Industrial Technology, Xuzhou, Jiangsu, P. R. China; 3 College of Materials and Chemical Engineering, West Anhui University, Luan, Anhui, P. R. China; 4 School of Chemistry and Molecular Engineering, East China University of Science and Technology, Shanghai, P. R. China; Parul University Parul Institute of Technology, INDIA

## Abstract

To address the demand for rapid detection of trace metaldehyde pesticide residues in agricultural products such as Dendrobium officinale Kimura et Migo (D. officinale), this paper proposes and validates a high-sensitivity metal-graphene hybrid metamaterial sensor, which exhibits significant advantages in the field of pesticide residue detection. The sensor consists of a quartz substrate, periodic gold array, polyimide (PI) spacer layer, and few-layer graphene, and is fabricated using standard micro-nano processing technologies. Utilizing a terahertz time-domain spectroscopy (THz-TDS) system, the reflection spectra of the sensor in response to metaldehyde solutions with different concentrations (0–2400 pg mL^-1^) were measured in the 0.6–1.5 THz frequency band. The results indicate that compared with the bare metamaterial, the introduction of graphene significantly enhances the sensor’s sensitivity, and its resonance intensity decreases noticeably with the increase in metaldehyde concentration. Simulation analyses reveal the existence of the toroidal dipole resonance mode and the mechanism by which the graphene layer induces a resonant red shift by increasing the effective capacitance, while confirming that the regulation of graphene’s Fermi energy level can dynamically modulate the resonance intensity. Experimental measurements show that the hybrid metamaterial sensor achieves a minimum detection limit of 100 pg mL^-1^ for metaldehyde, with a rapid response in the low concentration range (0–200 pg mL^-1^). This high sensitivity is primarily attributed to the effective modulation of the dielectric environment on the graphene surface by the monolayer adsorption of analytes at low concentrations. The sensor developed in this study exhibits excellent performance, providing an effective technical solution for the rapid screening of pesticide residues in the safety inspection of agricultural products such as D. officinale.

## Introduction

D. officinale is the dried stem of the perennial herb of the genus Dendrobium in the orchid family, which has been included in different editions of the Chinese Pharmacopoeia as a traditional, valuable, and nourishing medicinal herb in China [1–3]. D. officinale has been widely used in medicinal use and food ingredient for thousands of years, and is widely distributed in southern China. Studies have shown that different parts (leaves, stems, flowers) of D. officinale are rich in polysaccharides, alkaloids, stilbenoids, flavonoids, amino acids, trace elements, and other medicinal active ingredients beneficial to human health, and have significant pharmacological activities such as anti-inflammatory, antibacterial, antioxidant, anti-aging, anti-tumor, immunomodulatory, hypotensive, blood glucose regulation effects, etc., and are widely used in disease treatment, disease prevention, nutrition health care, delaying ageing, beautifying skin, and other fields [4–10]. Due to the low reproduction rate and slow growth of D. officinale in its natural growth environment, coupled with over-exploitation, wild D. officinale has been listed in the National Key Protected Wild Medicinal Species List and the Convention on International Trade in Endangered Species of Wild Fauna and Flora (CITES). As a result, wild D. officinale can no longer meet the needs of market development [9]. Consequently, cultivated sources constitute the predominant supply in commercial markets. However, during the cultivation of D. officinale, this crop is highly susceptible to pest and disease infestation, which directly impacts its yield and quality [9,11]. Snails represent one of the most prevalent pests affecting D. officinale, primarily occurring during the seedling growth stage. Wounds caused by snail feeding facilitate the invasion of pathogenic bacteria, inducing plant diseases. Furthermore, the white mucous secretions and green, string-like excrement left behind after snail crawling significantly impede the growth of D. officinale [12,13]. This pest is characterized by explosive outbreaks and extensive damage, capable of causing severe losses within a short period. To mitigate the impact of pests and diseases, an integrated pest management (IPM) strategy combining physical control, biological control, and chemical control is commonly employed [14]. Among the chemical options, metaldehyde is a highly effective, low-toxicity, and low-residue pesticide specifically targeting snails and slugs. However, the Maximum Residue Limit (MRL) standard for metaldehyde on D. officinale is critical for ensuring its safe application [15]. Therefore, establishing non-destructive detection methods for determining the MRL of metaldehyde in D. officinale has become an urgent research priority.

Conventional pesticide residue detection relies on chromatographic techniques such as Gas Chromatography (GC), Thin-Layer Chromatography (TLC), Gas Chromatography-Mass Spectrometry (GC-MS), High-Performance Liquid Chromatography (HPLC), and Liquid Chromatography-Mass Spectrometry (LC-MS) [16–18]. Although these well-established methods achieve exceptionally low detection limits, their application in pesticide residue analysis faces significant limitations, including a restricted scope of detectable pesticides, cumbersome sample pre-treatment procedures, and high operational costs. These constraints impede their efficiency and accessibility in practical scenarios [19]. Consequently, rapid detection methods-such as fluorescence colorimetry (FC), surface-enhanced Raman spectroscopy (SERS), near-infrared spectroscopy (NIRS), and surface plasmon resonance (SPR)-are rapidly emerging [20–24]. While these spectroscopic techniques offer advantages including rapid analysis and simplified sampling protocols, their accuracy and practicability remain insufficient to meet the demands of quantitative analysis and real-world application scenarios [19,25]. In recent years, Terahertz (THz) waves, electromagnetic radiation spanning 0.1 to 10 THz, have demonstrated unique advantages in identifying the fingerprint spectra of pesticide residues due to their low photon energy, penetration through non-polar materials, and matching with molecular vibrational-rotational transitions [26,27]. However, conventional THz technology faces inherent limitations in detecting trace pesticide residues: on the one hand, the concentration of pesticide residues typically range from parts per million (ppm) to parts per billion (ppb), resulting in characteristic absorption signals prone to obscuration by noise; on the other hand, the strong absorption of water in agricultural products (with an absorption coefficient of water as high as 220 cm ⁻ ¹ in the THz band) severely interferes with the identification of pesticide characteristic peaks [28,29]. As artificially engineered arrays of subwavelength structures, Metamaterials regulate the interaction between THz waves and target analytes through local field enhancement and high-Q resonances, thus becoming a pivotal technology to break through these bottlenecks [30]. The integration of these approaches to form THz metamaterial sensors is promoting the development of pesticide residue detection towards high sensitivity, portability, and practicality [30]. For example, Xu et al. developed a biosensing platform by incorporating a monolayer of graphene into a THz metamaterial absorber cavity, which displayed exceptional sensitivity with the detection of chlorpyrifos methyl at concentrations as low as 0.2 ng [31]. Aruna et al. proposed a design scheme of a THz metamaterial sensor based on graphene with a peak sensitivity reaching 22.75 GHz/refractive index unit (RIU) [32]. Lang et al. designed a graphene-based metamaterial biosensor, and the minimum detection concentration for phosalone in organophosphorus insecticides was 0.01 μg mL^-1^ [33]. Yan et al. proposed and experimentally validated a Fano resonance metamaterial-based silver nanoparticles sensor, which can detect acetamiprid solutions at a concentration as low as 100 pg mL^-1^ [34]. Among these remarkable research achievements, graphene, owing to its exceptional electrical and optical properties (such as ultrahigh carrier mobility, broadband optical responsiveness, and atomic-level thickness), has been integrated into THz metamaterial sensors [33]. When pesticide residue molecules adsorb onto the graphene surface, significant interfacial physicochemical interactions occur, including charge transfer, molecular dipole moment effects, and π-π stacking [35]. These interactions directly perturb the carrier concentration and distribution in graphene, thereby leading to a shift in its Fermi level. The alteration of the Fermi level profoundly influences the electromagnetic response characteristics of graphene in the THz frequency band, such as resonant frequency shift, resonant intensity variation, and phase alteration, which substantially enhances the detection sensitivity and specificity of the sensor for pesticide residues [35].

In this work, a THz hybrid graphene-metamaterial sensor was proposed, which features a metallic periodic pattern structure. The metamaterial sensor was fabricated with high precision using standard lithography and thermal evaporation techniques [36]. The transmission spectra were measured by a THz-TDS system. The reflection spectra of metaldehyde aqueous solutions with different concentrations (0–24,000 pg mL^-1^) drip-dried onto metamaterials with/without the few-layer graphene were investigated in the 0.6–1.5 THz frequency range. The experimental results revealed that the sensor exhibited excellent sensing performance by detecting the concentration of metaldehyde aqueous solutions. As the concentration of metaldehyde increased, the resonance intensity of the bare metallic metamaterial sensor changed little, whereas that of the hybrid graphene-metallic metamaterial sensor decreased significantly. The design of the metal-graphene hybrid metamaterial and its toroidal resonance characteristics enable this sensor to detect metaldehyde at concentrations as low as 100 pg mL^-1^. The primary function of this sensor is to detect the concentration of a single pesticide (e.g., metaldehyde in D. officinale) rather than separate various substances from mixed solutions. It can serve as a reusable and rapidly responsive pesticide screening device for real-time monitoring of pesticide contamination, thereby contributing to ensuring the quality of agricultural products and food safety. Additionally, this sensor is expected to promote safer development in large-scale agricultural industrialization.

## Structure design and experimental setup

**Fig 1:**
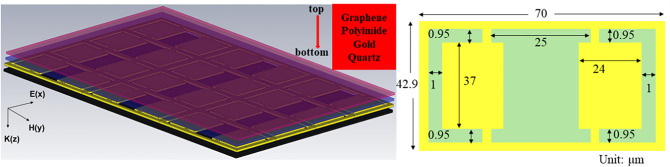
Schematic of the proposed metamaterial sensor and unit cell with dimensions.

**Fig 2:**
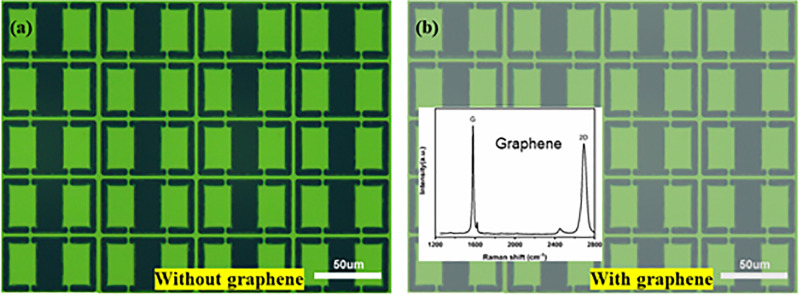
Optical microscope images of the fabricated samples (a) Without graphene and (b) with graphene (Inset: Raman spectroscopy of the graphene layer).

Fig 1 illustrates the local schematic of the proposed metal-graphene hybrid metamaterial sensor. The sensor consists of a 4 × 3 unit cell array, which is structured into four distinct layers from top to bottom: few-layer graphene, polyimide dielectric layer (thickness of 3 μm, relative permittivity ~3.2 at 1 GHz), periodically patterned gold array (thickness of 200 nm, conductivity of 4.561 × 10^7^ S m^-1^), and quartz substrate (thickness of 300 μm). Meanwhile, the arrows denote the detailed geometric parameters of the unit cell within the metamaterial periodic array. The metamaterial sample without graphene was successfully fabricated using standard lithography and thermal evaporation processes [37], as shown in Fig 2a. Based on this structure, a 3 μm thick polyimide (PI) film was deposited on the surface of the periodically patterned metal array layer via spin-coating technique. Graphene was prepared by chemical vapor deposition and transferred onto the PI film surface (with a size of 10 mm × 10 mm, using poly (methyl methacrylate) (PMMA) as the transfer support layer), ultimately constructing the hybrid graphene-metamaterial sensor, as shown in Fig 2b. The quality of graphene (e.g., presence of defects or impurities) can affect the periodic structure of the metamaterial or increase the contact resistance. High-quality graphene, due to its low defect density and high purity, is more conducive to effectively regulating electromagnetic response, thereby improving the detection limit and sensitivity of the sensor. Raman spectroscopy analysis was performed on the transferred graphene layer in the sensor structure (excitation wavelength of 514 nm, as shown in the inset of Fig 2b), and the intensity ratio of the characteristic G band (located at ~1580 cm^-1^) to 2D band (located at ~2680 cm^-1^) (I_G_/I_2D_) was 1.44, indicating that it is high-quality few-layer graphene [36].

**Fig 3:**
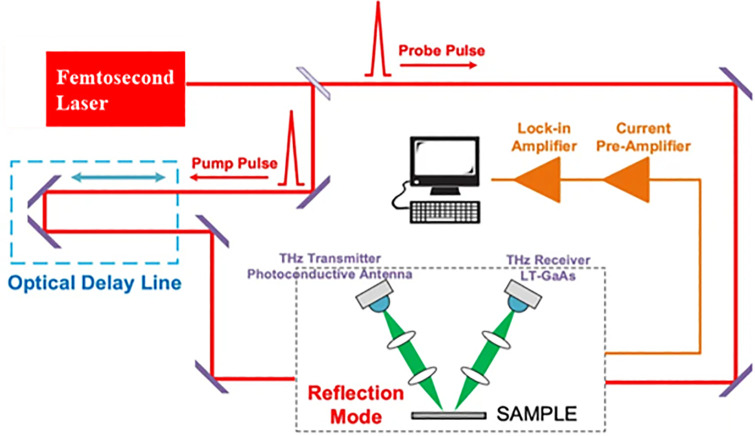
Schematic diagram of the THz TDS system in the experiment.

The resonance characteristics of the metal-graphene hybrid metamaterial samples were measured using a commercial THz-TDS (TAS7500TS, Advantest Corporation) system, with a schematic shown in Fig 3. This THz-TDS system employs an LED-pumped all-fiber Femtosecond laser to generate THz pulses. The laser operates at a wavelength of 1550 nm, with a pulse duration of 100 fs and a repetition frequency of 100 MHz. During experimental measurements, the laser power was set to 100 mW, and the laser beam was split into two identical beams. One beam generates THz pulses via a GaAs-biased photoconductive antenna, while the other serves as a detector for THz pulses, generating and probing THz waves through asynchronous sampling. Dry air was introduced to maintain a stable environment within the testing system. The temperature in the THz-TDS setup was 24 ± 1 °C, and the humidity was controlled below 1%. The THz time scan was 40 ps, with a spectral resolution of 40 GHz. The THz frequency range measured by the TAS7500TS is approximately 0.1 to 4 THz, with a resolution of 1.9 GHz and fast THz scan of 16 ms. The signal-to-noise ratio (SNR) was exceeded 45 dB with an average of 1024 scans measurements. Each sample at the analyzed concentration was subjected to at least 15 parallel determinations to minimize errors. Time-domain signals were converted into frequency-domain signals via the Fast Fourier Transformation (FFT) during signal processing. The reflection spectra were calculated according to the formula $R(\omega )={\left|\frac{E_{s}(\omega )}{E_{r}(\omega )}\right|}^{\text{2}}$ with a gold mirror reference of approximately 100% reflectivity, where $E_{s}(\omega )$ represents THz signals reflected from the metamaterial sample, and $E_{r}(\omega )$ denotes THz signals of gold mirror reference. Metaldehyde (C₈H₁₆O₄, CAS No.: 108-62-3, purity 98%) was purchased from Sinopharm Chemical Reagent Co., Ltd. A 100 mg L^-1^ stock solution was prepared by accurately weighing 10 mg of metaldehyde and dissolving it in 100 mL of deionized water, which was stored at 4 °C in the dark. The stock solution was then diluted to prepare eight solutions with different concentrations: 100, 200, 300, 400, 700, 1000, 1500, and 2400 pg mL^-1^. For measurement, 20 μL of each solution was dropped onto the surface of the metal-graphene hybrid metamaterial sensor and dried in air before testing. The preparation procedure for the 100 pg mL^-1^ metaldehyde solution is provided in the Supporting Information.

## Results and discussion

The designed metal-graphene hybrid metamaterial structure was modeled and numerically analyzed using the commercial simulation software CST Microwave Studio based on the Finite Integration Technique (FIT), which was designed to model the electromagnetic resonance characteristics of the metamaterial, the modulation effect of the graphene Fermi energy level, and the toroidal dipole mode. The theoretical basis of FIT, proposed by Professor Thomas Weiland in 1977, is rooted in spatial discretization methods [38]. The core of this method is to replace the time derivative term with the central difference iterative solution. In this paper, adaptive mesh refinement was employed for grid generation. After establishing the unit cell model, the simulation boundary conditions were set as follows: magnetic and electric field boundary conditions were applied in the x- and y-directions to simulate an infinitely large array, respectively, while an open boundary condition was specified in the z-direction to Simulate free space. The frequency-domain solver was utilized to simulate the constructed unit cell model, enabling the acquisition of metamaterial unit structures with specific electromagnetic responses. The convergence accuracy of the frequency-domain solver was set to 1 × 10 ⁻ ¹².

In these simulations, quartz with a relative permittivity of ε = 4.41 + 0.004i was used as the substrate, while the metallic array was designed using gold with an electrical conductivity of 4.561 × 10⁷ S/m [39]. The skin depth of the gold layer typically ranges from several nanometers to several tens of nanometers, rendering it highly effective in absorbing incident electromagnetic waves and making it exceptionally suitable for application as a reflective layer. In studies on terahertz sensors and tunable devices, the Kubo model has been employed to describe the relationship between the Fermi level and the conductivity of graphene, with the Kubo formula being widely used to calculate the dynamic conductivity of graphene [40]. Within the terahertz frequency range, the conductivity of graphene is primarily influenced by intraband carrier transitions [41]. The Kubo model can be expressed as follows [42]:


$$\sigma \left(\omega ,\;\Gamma ,\;\mu_{c},\;T\right)=\;\sigma_{inter}\left(\omega ,\;\Gamma ,\;\mu_{c},\;T\right)+\;\sigma_{intra}\left(\omega ,\;\Gamma ,\;\mu_{c},\;T\right)\;\;\;\;\;$$ (1)

$$\sigma_{inter}\left(\omega ,\;\Gamma ,\;\mu_{c},\;T\right)=\;\frac{ie^{\text{2}}(\omega +i\text{2}\Gamma )}{\pi \hbar^{\text{2}}}\;\int_{\text{0}}^{\infty }\frac{f_{d}(-\xi )-f_{d}(\xi )}{(\omega +i\text{2}\Gamma {)}^{\text{2}}-\text{4}{\left(\frac{\xi }{\hbar }\right)}^{\text{2}}}d\xi \;\;\;\;\;\;\;$$ (2)

$$\sigma_{intra}\left(\omega ,\;\Gamma ,\;\mu_{c},\;T\right)=\;\frac{-ie^{\text{2}}}{\pi \hbar^{\text{2}}(\omega +i\text{2}\Gamma )}\;\int_{\text{0}}^{\infty }\xi (\frac{{\partial f}_{d}(\xi )}{\partial \xi }-\;\frac{{\partial f}_{d}(-\xi )}{\partial \xi })d\xi \;\;\;\;\;\;\;$$ (3)

$$f_{d}(\xi )=\;\frac{\text{1}}{\text{exp}\left(\left(\xi -\;\mu_{c}\right)/\left(k_{B}T\right)\right)+\text{1}}\;\;\;\;\;\;\;\;\;$$ (4)

Here, $\mu_{c}$ is the chemical potential of graphene or the Fermi energy level of graphene, ω is the angular frequency of the photon, Γ is the scattering rate, T is the ambient temperature, $f_{d}(\xi )$ is the Fermi-Dirac distribution, e is the electron charge, ℏ is the reduced Planck constant, and ξ is the photon energy. The relationship between the chemical potential and carrier concentration of graphene is as follows [43]:


$$\mu_{c}=\;\hbar \nu_{F}{\left(\pi n\right)}^{\text{1}/\text{2}}$$ (5)

$$n=\;\varepsilon_{\text{0}}\varepsilon V_{g}/ed$$ (6)

Among them, n denotes the graphene carrier concentration, $\nu_{F}$ (1 × 10^6^ m s^-1^) is the Fermi velocity of graphene, $V_{g}$ signifies the bias voltage applied to the graphene layer, $\varepsilon_{\text{0}}$ and ε are the dielectric constants of vacuum and insulating substrate materials, respectively, and d represents the thickness of the insulating layer material. As indicated by the expression, the magnitude of $\mu_{c}$ primarily depends on the carrier concentration in graphene. By adjusting $V_{g}$, one can modulate the graphene carrier concentration n, thereby enabling dynamic modulation of the graphene conductivity. In CST Microwave Studio, the electromagnetic model of multilayer graphene is rigorously defined through these physical parameters, and the thickness of graphene is generally set at 1 nm (equivalent to the thickness of ten layers of monolayer graphene) instead of the actual thickness of 0.335 nm, mainly for the convenience of calculation and to save computer memory and simulation time. Simulations were performed using the Frequency Domain Solver. The boundary conditions were configured with an incident Terahertz wave exhibiting linear polarization along the y-direction and propagating along the z-direction (as defined in Fig 1).

**Fig 4:**
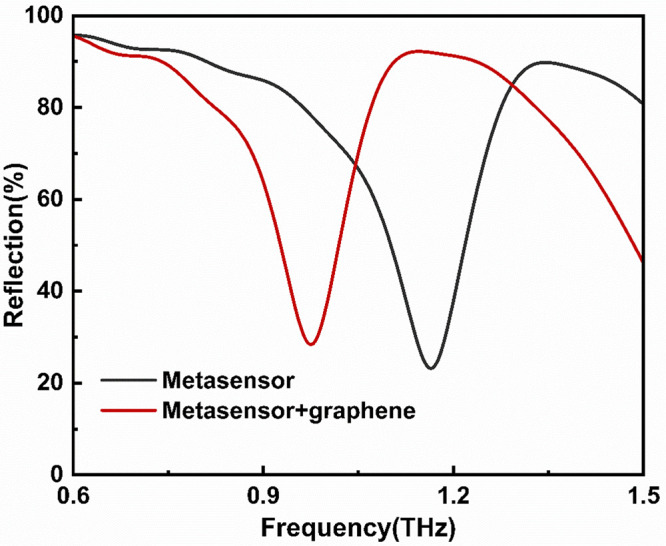
The simulated reflection spectra of metamaterial sensors with/without graphene.

**Fig 5:**
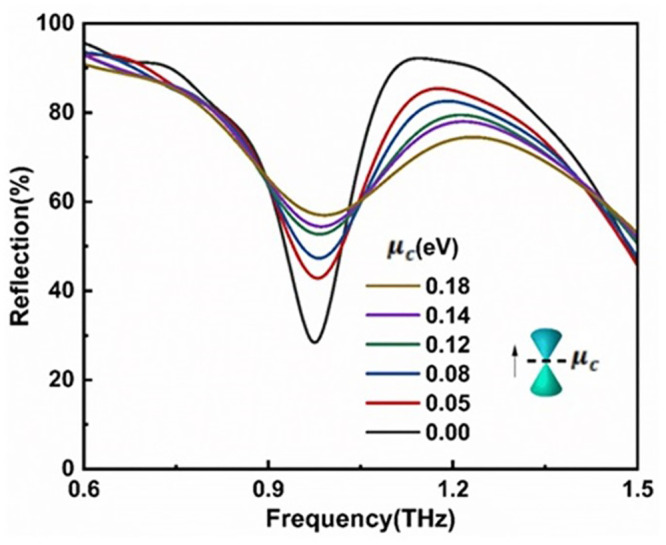
The simulated reflection spectra at 1.136 THz for metal-graphene hybrid metamaterial sensor as a function of the Fermi energy level (0-0.18 eV).

**Fig 6:**
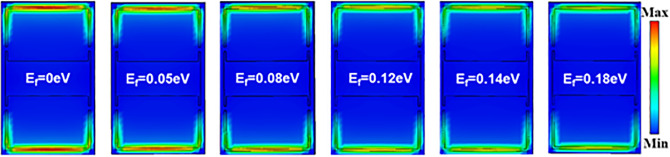
The simulated electric field distribution at 1.136 THz for metal-graphene hybrid metamaterial sensor as a function of the Fermi energy (0-0.18 eV).

To elucidate the underlying resonance mechanisms of the designed metal-graphene hybrid metamaterial structure, numerical simulations of the metamaterial’s transmission characteristics were conducted. The spectral results obtained using a terahertz photonic heterodyne frequency-domain spectrometer are illustrated in Fig 4, where the reflection spectra of the bare metamaterial without graphene and the graphene-integrated metamaterial are represented by black and red lines, respectively. The simulation results demonstrate that within the frequency range of 0.6 THz to 1.5 THz (Resonance frequency band), the bare metamaterial without graphene exhibits a resonance peak at 1.329 THz and a distinct resonance dip at 1.164 THz. Upon depositing the few-layer graphene on the metamaterial surface, the dielectric constant distribution around the metallic metamaterial undergoes significant changes. According to the resonance frequency formula $\omega \;\propto \text{1}/\sqrt{L_{m}C_{eff}}\;$ ($L_{m}$ represents the equivalent inductance of the metallic structure, $C_{eff}$ denotes the equivalent capacitance), the introduction of graphene layers forms additional capacitive coupling at the graphene-metal interface, leading to an increase in $C_{eff}$. This directly results in a decrease in the resonance frequency, manifesting as a red shift [31,44]. Furthermore, an increase in the number of graphene layers further augments $C_{eff}$, thereby enhancing the red shift magnitude [31]. As illustrated in Fig 4, the resonance frequencies red shift to 1.136 THz and 0.978 THz, respectively. By modulating the Fermi level ($\mu_{c}$) of graphene within the range of 0–0.15 eV through electrostatic gating, we systematically investigated the dynamic evolution of electromagnetic resonance characteristics in terahertz metamaterial (as illustrated in Fig 5). Simulation results demonstrate that as $\mu_{c}$ increases from 0 eV to 0.15 eV, the resonance intensity significantly diminishes, a phenomenon closely correlated with the cross-sectional electric field distribution at the 1.136 THz frequency point (Fig 6) [45,46]. At $\mu_{c}$= 0, the electric field distribution exhibits strong localized enhancement effects at the edges of the metallic microstructures, characteristic of typical Surface Plasmon Resonance (SPR) modes [47]. However, when $\mu_{c}$ rises to 0.15 eV, the previously observed electron hotspots gradually disappear [48]. This resonance suppression effect originates from the modulation of graphene conductivity by the elevated Fermi level: according to the Kubo formula, an increase in $\mu_{c}$ significantly enhances the intra-band conductivity ($\sigma_{intra}$), thereby intensifying the ohmic losses of electromagnetic waves in the graphene layer and ultimately weakening the coherence of plasmon oscillations [45,49,50]. The tunability of graphene’s Fermi level ($\mu_{c}$) through chemical doping enables terahertz metal-graphene hybrid metamaterial to exhibit exceptional sensitivity to external stimuli at the Dirac point ($\mu_{c}$ ≈ 0 eV). By integrating the specific response of terahertz bands to molecular fingerprint spectra with the localized field enhancement effect of metamaterial, this approach provides an innovative solution for agricultural product safety monitoring.

**Fig 7:**
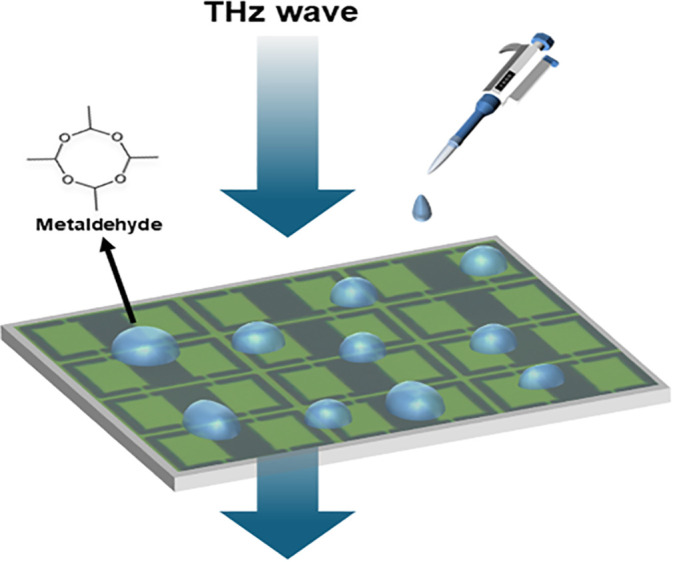
Schematic diagram of the metaldehyde sensing measurement.

**Fig 8:**
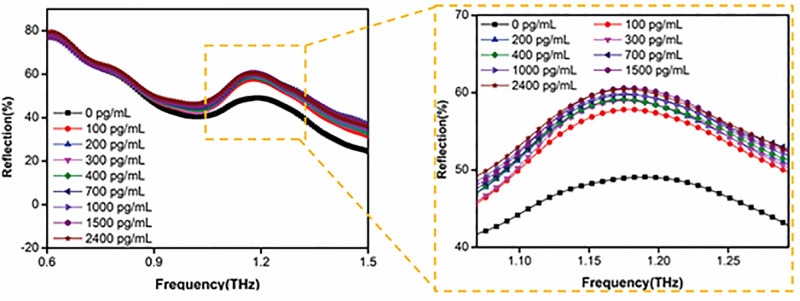
The experimental measurement of reflection spectra of the designed metal-graphene hybrid metamaterial sensor under different concentrations of metaldehyde solutions (the dotted box is a partial enlarged view).

To evaluate the performance of the fabricated metal-graphene hybrid metamaterial terahertz biosensor, experiments were conducted using gradient concentrations of metaldehyde aqueous solutions (100, 200, 300, 400, 700, 1000, 1500, and 2400 pg mL^-1^). A precise volume of 20 μL of each sample was dispensed onto the surface of metal-graphene hybrid metamaterial sensor using a micropipette, followed by incubation at room temperature until complete evaporation of the solvent (water) to eliminate strong absorption interference of terahertz signals by water. After the sensor surface was completely dried, the reflection spectra were measured using a THz-TDS system. The THz wave is incident perpendicularly along the Z-axis (θ = 0°) onto the device surface, and the schematic diagram of the sensing measurement is illustrated in Fig 7. The relationship between reflectivity (R) and reflection coefficient ($S_{\text{1}\text{1}}$) is $R=\;{\left|S_{\text{1}\text{1}}\right|}^{\text{2}}$, where S is the parameter simulated in CST Microwave Studio. As the drip-dried concentration of metaldehyde increased from 0 to 2400 pg mL^-1^, the reflection spectra of the metal-graphene hybrid metamaterial sensor are illustrated in Fig 8. The reflectivity (approximately 49%) of the metal-graphene hybrid metamaterial in the absence of analytes is determined by the intrinsic carrier properties of graphene (such as carrier density of approximately 10^12^ cm^-2^ and conductivity) and the electromagnetic resonance characteristics of the metamaterial [51]. Under these conditions, the incident electromagnetic wave undergoes partial reflection and transmission at the graphene-metal interface, while the delocalized π-electron system of graphene remains undisturbed. The electromagnetic coupling and energy dissipation of the metamaterial are in equilibrium, resulting in a baseline level of reflected signal [52]. When the concentration of metaldehyde is 100 pg mL^-1^, the reflectivity of the metal-graphene hybrid metamaterial increases to approximately 58%, which is mainly attributed to two reasons. Firstly, dielectric constant modulation: When metaldehyde molecules are adsorbed on the graphene surface at a low concentration (pg mL^-1^), the ordered monolayer adsorption of metaldehyde molecules (containing polar C-O-C bonds) modifies the local dielectric environment on the graphene surface. The ordered arrangement of the monolayer molecules leads to an increase in the real part of the effective permittivity ($\varepsilon_{eff}$) in the near-field region of the metamaterial [53,54]. According to Fresnel’s reflection law, the interface reflectivity is related to the impedance matching degree between the media on both sides [55]. The change in $\varepsilon_{eff}$ improves the impedance matching between the metamaterial and free space, reduces transmission loss, enhances the energy confinement capability of the resonance mode, and thus results in a significant increase in reflectivity (from 48% to 58%) [55]. Secondly, weak charge transfer effect: Metaldehyde is a neutral molecule, and the interaction with the delocalized π electrons of graphene is dominated by van der Waals forces or dipole-dipole interactions, with extremely weak charge transfer effect [56,57]. Therefore, it does not significantly alter the carrier density and conductivity of graphene, which avoids the aggravation of resonance loss caused by enhanced carrier scattering and ensures the efficient excitation of the metamaterial resonance mode [58]. This is consistent with the results obtained by Li et al. through density functional theory calculations of the charge transfer amount between neutral molecules and graphene [58].

However, when the concentration of metaldehyde is further increased from 100 pg mL^-1^ to 2400 pg mL^-1^, the reflectivity of the metal-graphene hybrid metamaterial sensor only slightly increases from approximately 58% to about 61%, with an increment of merely around 3%. This phenomenon is mainly attributed to two mechanisms:

Firstly, the number of adsorption sites on the graphene surface (such as defects, edges, and specific functional groups) is limited (consistent with the results in Fig 2). When the concentration rises to 2400 pg mL^-1^, the monolayer adsorption on the surface approaches a saturated state (in line with the characteristics of the Langmuir adsorption isotherm), and excess molecules are adsorbed on top of the monolayer through multi-layer stacking [59,60]. Since the resonant field of the metamaterial is mainly concentrated within the nanoscale range of the surface, the outer-layer molecules, being far from this strong-field region, exhibit a significantly attenuated ability to modulate the effective permittivity ($\varepsilon_{eff}$) [61]. This leads to a minimal incremental change in the dielectric environment, thereby limiting the increase in reflectivity.

Secondly, multi-layer adsorption at high concentrations may be accompanied by an increase in the disorder of molecular arrangement (e.g., randomization of stacking directions), forming a scattering layer with an inhomogeneous distribution of dielectric constants. Such structural disorder can induce additional scattering losses of electromagnetic waves, partially offsetting the reflectivity enhancement effect brought by dielectric modulation and further suppressing the increment of reflectivity [62,63].

**Fig 9:**
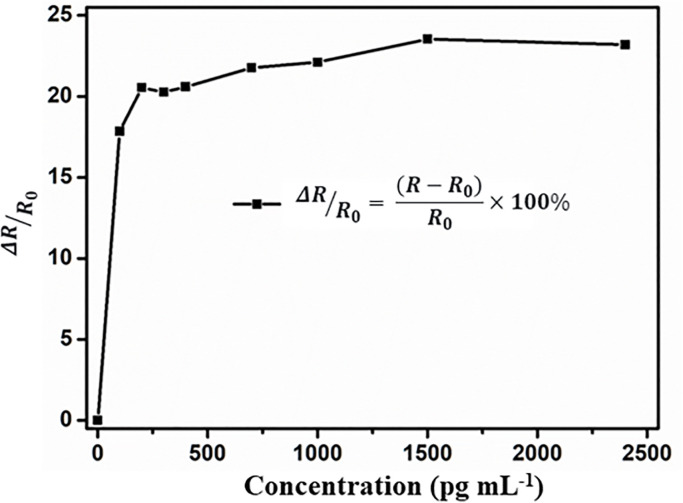
The measured differential reflection $\frac{\Delta R}{R_{\text{0}}}\;$ as a function of pesticide concentrations.

To quantify the performance of the metal-graphene hybrid metamaterial sensor, Fig 9 plots the differential reflectivity of the sensor at the resonance peak, with its expression given by $\frac{\Delta R}{R_{\text{0}}}=\;\frac{\left(R-R_{\text{0}}\right)}{R_{\text{0}}}\times \text{1}\text{0}\text{0}\%$, where R represents the terahertz pulse reflectivity induced by chemical doping at a specific pesticide concentration, and $R_{\text{0}}$ denotes the terahertz reflectivity in the absence of pesticide solution. Fig 9 shows that the relative reflectivity change curve of the proposed metal-graphene hybrid metamaterial sensor exhibits a significant concentration dependence: in the low concentration range (0 ~ 200 pg mL^-1^), $\frac{\Delta R}{R_{\text{0}}}$ rises rapidly; as the concentration further increases, the increment gradually slows down; when the concentration reaches 1500 pg mL^-1^, $\frac{\Delta R}{R_{\text{0}}}$ tends to saturate and finally stabilizes at approximately 24%. This result is consistent with the conclusion in Fig 8, collectively indicating that the detection limit of the prepared metal-graphene hybrid metamaterial sensor can be as low as 100 pg mL^-1^. Additionally, the surface current density and magnetic field distribution of the metamaterial at the resonant frequency of 1.136 THz are shown in S1 Fig. It can be clearly observed that currents flowing in opposite directions (indicated by red arrows) appear on the metallic rings, while oppositely oriented magnetic fields are excited around the intermediate metallic strips between adjacent unit cells. Furthermore, a unique closed-loop magnetic field configuration forming a head-to-tail connection (blue arrows) emerges around the long metallic bars of the unit cells. This structure visually suggests the excitation of a toroidal dipole resonance (purple arrows) in the metamaterial sensor. Such a toroidal dipole resonance mode exhibits unconventional optical properties, including low radiative loss, small mode volume, and strong electromagnetic field confinement, which constitutes another key mechanism enabling the ultra-sensitive detection of metaldehyde solution [64].

Compared with the achievements of other research teams reported in the literature (as shown in Table 1), the metal-graphene hybrid metamaterial sensor proposed in this paper exhibits superior performance in terms of the detection limit of pesticide concentration.

**Table 1:** The performance comparison of pesticide sensing by THz sensors with previous work.

Year	Sensor structure	Analyte	Limit of detection	References
2019	graphene/metamaterial	chlorpyrifos-methyl	0.2 ng mL^-1 ^	[31]
2020	CNT/metamaterial	2,4-dichlorophenoxyacetic	10 ng	[65]
2021	metasurfaces (WIT)	midkine	0.5μg mL^-1 ^	[66]
2022	N-CNTs/Au NPs	nereistoxin-related insecticides (NRIs)	1.33 μg/L	[67]
2022	graphene/ metamaterial/perovskite	whey protein	6.25 ng mL^-1 ^	[68]
2023	graphene/metamaterial	phosalone	0.01μg mL^-1^	[33]
2024	graphene/metamaterial	chlorpyrifos-methyl	10 ppm	[35]
2025	graphene/metamaterial	metaldehyde	100 pg mL^-1^	This work

## Conclusion

In summary, a metal-graphene hybrid metamaterial sensor was successfully designed, fabricated, and characterized for highly sensitive detection of the pesticide metaldehyde. Using a THz-TDS system, the reflective spectral responses of the sensor to metaldehyde aqueous solutions with different concentrations (0–2400 pg mL^-1^) were systematically investigated in the 0.6–1.5 THz frequency band. Simulation analysis revealed that this structure can excite the toroidal dipole mode; the introduction of graphene induces a redshift in the resonance frequency by increasing the effective capacitance, and the Fermi level of graphene (regulated via chemical doping) can dynamically modulate the resonance intensity. Experimental results showed that in the low concentration range (0–200 pg mL^-1^), the relative reflectivity change (ΔR/R₀) of the sensor increased rapidly, and a stably detectable signal was generated at a concentration of 100 pg mL^-1^, confirming that its detection limit is as low as 100 pg mL^-1^. This high sensitivity stems from the modulation effect of the ordered monolayer adsorption of metaldehyde molecules on the local dielectric environment of the graphene surface at low concentrations, a process that improves the impedance matching between the metamaterial and free space. In contrast, the saturation of adsorption at high concentrations (consistent with the characteristics of the Langmuir adsorption isotherm) and the scattering losses caused by disordered molecular stacking collectively limit the increment of the response. The sensor exhibits excellent sensing performance with the potential for reusability and rapid response, providing an effective technical solution for real-time monitoring of pesticide contamination and ensuring the safety of agricultural products.

## Supporting information

S1 Fig The surface current density and magnetic field distribution of the metamaterial at the resonant frequency of 1.136 THz. (TIF)

S1 File The preparation of 100 pg mL^-1^ metaldehyde solution. The original experimental data of reflection spectra of the designed metal-graphene hybrid metamaterial sensor under different concentrations of metaldehyde solutions. (WinRAR).
(PDF)
