# Supplementary material for: A terahertz graphene-metamaterial sensor for highly sensitive detection of trace pesticide metaldehyde
Source: PLoS One. 2025 Dec 12;20(12):e0338331. doi: 10.1371/journal.pone.0338331 (PMC12700456; doi:10.1371/journal.pone.0338331)
Supplement: S1 File — The original experimental data of reflection spectra of the designed metal-graphene hybrid metamaterial sensor under different concentrations of metaldehyde solutions. (WinRAR). (PDF) [file pone.0338331.s002.pdf]

Supporting Information

A Terahertz Graphene-Metamaterial Sensor for Highly Sensitive Detection of Trace Pesticide Metaldehyde

Dazhi Zhang[a,b], Xinlei Ruan[c], Maosheng Yang[c], Honglai Liu[d], Xiaobing Li[a,*], Peipei Li[c,*]

[a] School of Chemical Engineering & Technology, China University of Mining and Technology, Xuzhou 221116, Jiangsu, P. R. China.

[b] Xuzhou College of Industrial Technology, Xuzhou 221114, Jiangsu, P. R. China.

[c] College of Materials and Chemical Engineering, West Anhui University, Luan 237012, Anhui, P. R. China.

[d] School of Chemistry and Molecular Engineering, East China University of Science and Technology, Shanghai 200237, P. R. China.

Corresponding author: Xiaobing Li, Peipei Li

E-mail address: Xiaobing.li@cumt.edu.cn; lpp0516@126.com

The 100 pg mL^{-1} metaldehyde solution was prepared through a serial dilution process as follows: Exactly 1000 μL of the 100 mg mL^{-1} stock solution was pipetted into a 100 mL volumetric flask, which was then brought to volume with ultrapure water to yield a 10 mg mL^{-1} metaldehyde intermediate solution. Next, exactly 100 μL of the first intermediate solution (10 mg mL^{-1}) was pipetted into a new 100 mL volumetric flask and diluted to volume with ultrapure water to obtain a 10 μg mL^{-1} metaldehyde intermediate solution. Finally, exactly 1000 μL of the second intermediate solution (10 μg mL^{-1}) was pipetted into a final 100 mL volumetric flask and made up to volume with ultrapure water to obtain the 100 pg mL^{-1} working solution. A method blank sample, prepared by subjecting ultrapure water to the identical dilution procedure, was analyzed concurrently with the final sample to account for and subtract any potential background interference.
